# Electrophysiological In Vitro Study of Long‐Range Signal Transmission by Astrocytic Networks

**DOI:** 10.1002/advs.202301756

**Published:** 2023-07-23

**Authors:** Nataly Hastings, Yi‐Lin Yu, Botian Huang, Sagnik Middya, Misaki Inaoka, Nadia A. Erkamp, Roger J. Mason, Alejandro Carnicer‐Lombarte, Saifur Rahman, Tuomas P. J. Knowles, Manohar Bance, George G. Malliaras, Mark R. N. Kotter

**Affiliations:** ^1^ Department of Clinical Neurosciences University of Cambridge Cambridge CB2 0QQ UK; ^2^ Wellcome‐MRC Cambridge Stem Cell Institute University of Cambridge Cambridge CB2 0AW UK; ^3^ Electrical Engineering Division Department of Engineering University of Cambridge Cambridge CB3 0FA UK; ^4^ Department of Neurological Surgery Tri‐Service General Hospital National Defence Medical Centre Taipei, Neihu District 11490 Taiwan; ^5^ Yusuf Hamied Department of Chemistry Centre for Misfolding Diseases University of Cambridge Lensfield Road Cambridge CB2 1EW UK; ^6^ Cavendish Laboratory Department of Physics University of Cambridge J J Thomson Ave Cambridge CB3 0HE UK

**Keywords:** astrocyte, calcium, electrophysiology, gap junction, syncytium

## Abstract

Astrocytes are diverse brain cells that form large networks communicating via gap junctions and chemical transmitters. Despite recent advances, the functions of astrocytic networks in information processing in the brain are not fully understood. In culture, brain slices, and in vivo, astrocytes, and neurons grow in tight association, making it challenging to establish whether signals that spread within astrocytic networks communicate with neuronal groups at distant sites, or whether astrocytes solely respond to their local environments. A multi‐electrode array (MEA)‐based device called AstroMEA is designed to separate neuronal and astrocytic networks, thus allowing to study the transfer of chemical and/or electrical signals transmitted via astrocytic networks capable of changing neuronal electrical behavior. AstroMEA demonstrates that cortical astrocytic networks can induce a significant upregulation in the firing frequency of neurons in response to a theta‐burst charge‐balanced biphasic current stimulation (5 pulses of 100 Hz × 10 with 200 ms intervals, 2 s total duration) of a separate neuronal‐astrocytic group in the absence of direct neuronal contact. This result corroborates the view of astrocytic networks as a parallel mechanism of signal transmission in the brain that is separate from the neuronal connectome. Translationally, it highlights the importance of astrocytic network protection as a treatment target.

## Graphical abstract

Astrocytes are brain cells that form large‐scale networks; the role of astrocytic coupling in information processing is not well understood. The designed AstroMEA device shows that astrocytic networks can transmit signals between independent groups of neurons+astrcoytes. This result has conceptual implications for modeling brain function and gives a new translational perspective on astrocytic networks as valuable therapeutic targets.

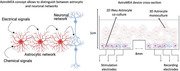

## 1 Introduction

Astrocytes represent a brain cell type with many important functions including potassium buffering,^[^ ^1^ ^]^ glutamate transport at the synapse,^[^ ^2^ ^]^ and chemical transmitter release^[^ ^3^ ^]^ that can contribute to the behavioral^[^ ^4^, ^5^, ^6^ ^]^ and sleep‐wake cycle^[^ ^7^, ^8^ ^]^ control. These cells are known to be non‐electrically excitable as opposed to neurons due to the linear relationship between voltage and current at astrocytic membranes which is related to the high extent of direct coupling among individual astrocytic cytoplasms via gap junctions creating as isopotential syncytium^[^ ^9^ ^]^ as these cells do not generate action potentials upon depolarization; at the same time there is an increasing appreciation of astrocytes being “electrically active” cells as they exhibit hyperpolarized membranes and express a number of voltage‐gated channels.^[^ ^10^ ^]^ The most well‐described mode of astrocytic signaling is calcium elevations, oscillations, and spreading waves that can be detected at cell bodies as well as processes.^[^ ^11^ ^]^ Calcium signals in astrocytes have been found to arise in response to chemical transmitters such as adenosine triphosphate (ATP), γ‐aminobutyric acid (GABA), glutamate, and dopamine;^[^ ^12^, ^13^ ^]^ mechanical stimulation;^[^ ^14^ ^]^ hyperosmolarity;^[^ ^15^ ^]^ depletion of extracellular calcium;^[^ ^16^ ^]^ or spontaneously.^[^ ^17^ ^]^ These astrocytic calcium events have been shown to influence neuronal signaling via triggering the release of chemical transmitters from astrocytes^[^ ^18^, ^19^, ^20^, ^21^ ^]^ that can induce long‐term potentiation;^[^ ^22^ ^]^ to induce proliferation of myelinating cells;^[^ ^23^ ^]^ and to adjust the level of blood flow to the brain.^[^ ^24^, ^25^, ^26^ ^]^ On a higher organismal level, reduction in calcium signaling in striatal astrocytes could lead to behavioral defects,^[^ ^5^ ^]^ while enhancing calcium signaling in hippocampal astrocytes was conducive to new memory acquisition.^[^ ^27^ ^]^ Calcium signals can spread between neighboring astrocytes via gap junctions—channels directly connecting cell cytoplasms of two cells and allowing for electrical coupling^[^ ^28^ ^]^ plus passage of small metabolites,^[^ ^29^ ^]^ as well as via chemical transmitter release followed by receptor engagement.^[^ ^30^ ^]^ The ability of astrocytes to form large‐scale communicating networks^[^ ^31^, ^32^ ^]^ has been reported; however, the full extent of the roles of astrocytic long‐range coupling and calcium wave spread in these cells (as opposed to the calcium events within individual astrocytes responding to their local environments) are still under investigation. Proposed functions of calcium transmission within astrocytic networks include propagation of injury signals to trigger stem cell proliferation,^[^ ^33^ ^]^ neuronal signaling synchronization,^[^ ^31^, ^34^, ^35^ ^]^ and memory formation via altering neuronal excitability and regulating synaptic plasticity^[^ ^36^ ^]^; at the same time, abnormal calcium waves have been associated with pathological states such as models of Alzheimer's disease.^[^ ^37^, ^38^ ^]^ Originally, calcium communication between astrocytes was considered too slow‐paced for information transfer relevant to human cognition—this notion has been challenged by recent observations of rapid astrocytic calcium responses comparable to the timescales of neuronal responses,^[^ ^39^ ^]^ which was advanced by novel technique development such as genetically encoded calcium indicators (GCaMPs) capable of detecting calcium signals at astrocyte processes in addition to the less frequent cell body events.^[^ ^40^ ^]^

In the current paper, we have hypothesized that astrocytic networks are capable of transferring long‐range signals that can change neuronal electrical behavior at distant sites away from an initial signal trigger location (in the brain this could be conceptualized as transferring information between separate nuclei with specific functions that somehow converge on complex coordinated cognitive perception and behavior—phenomenon termed as “binding problem”^[^ ^41^ ^]^). This signal trigger can be, for example, a period of enhanced electrical activity due to sensory stimulation^[^ ^25^ ^]^ that is a prerequisite for memory formation or behavioral response. These astrocytic network signals are likely to be closely associated with calcium waves given their key functions described in the literature, however, other less well‐studied modes of astrocytic communication such as electrical coupling and/or transmission of other second messengers may also contribute. If such astrocytic network‐exclusive transmission that can induce changes in neuronal signaling in response to distant electrical stimuli could be demonstrated, this would suggest an existence of a parallel cellular network in the brain that is capable of transferring as well as possibly modifying and integrating information arising from external and internal neuronal triggers as each human astrocyte can receive inputs from hundreds of thousands to millions of neuronal synapses,^[^ ^42^ ^]^ thus expanding our understanding of the computational complexity of the brain. This view is close to the “astrocentric hypothesis” of human consciousness proposed by James Robertson, who suggested the existence of such astrocytic networks integrating information from neuronal signals into a seamless conscious state^[^ ^43^, ^44^ ^]^—which however turned out difficult to test experimentally or apply to translational research to this date. While in vitro experiments using multi‐electrode arrays (MEAs) cannot model the complexities of consciousness, demonstrating the possibility of detection and transmission of signals exclusively within astrocytic networks which are capable of changing distant neuronal behavior would indicate that the roles of astrocytic networks need to be specifically considered while modeling the computational abilities of the brain since purely neuronal models would not be representative.

**Figure 1:**
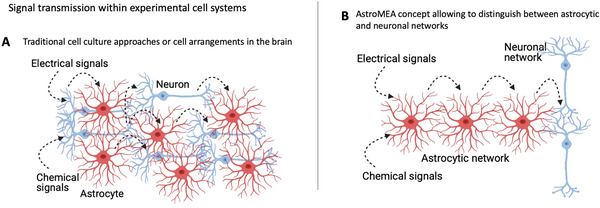
Schematic representation of the AstroMEA hypothesis. Red – astrocytes, blue – neurons.

The ability of neuronal fibers to transmit signals between distant regions of the brain or culture is easier to demonstrate due to the action potential propagation via long‐reaching axons (for instance, isolated neuronal groups can be bridged via microchannels where only axons extend), while the ability of astrocytic networks to do so have been harder to study. One technical problem in particular is distinguishing between the neuron‐to‐astrocyte‐to‐neuron signal propagation versus the propagation of signals within a network of astrocytes (astrocyte‐to‐astrocyte‐to‐neuron). Since neuronal axons are typically longer than astrocytic projections, limiting axonal growth while maintaining astrocytic connectivity is practically challenging. In co‐culture experiments as well as in slice cultures or in vivo, astrocytes and neurons are most often found in a tight mixture where both cell types may participate in information spread (**Figure** **1**).

While seemingly insignificant, conceptual differentiation between these scenarios (neuron‐to‐astrocyte‐to‐neuron signal propagation versus propagation of signals within a network of astrocytes) has important implications for basic science and translational therapy design. For instance, many current therapeutic approaches focus exclusively on neuronal support or neuronal function replacement, while astrocytic network restoration was not traditionally prioritized. To illustrate, differentiation of midbrain astrocytes into dopaminergic neurons was proposed as a treatment strategy for Parkinson's disease (PD)^[^ ^45^ ^]^—this approach would alleviate dopamine level decline in the short‐term, however long‐term effects of decreasing numbers of functional astrocytes on astrocytic signaling as well as neuronal function have not been explored. At the same time, accumulating body of literature suggests that the introduction of healthy astrocytes into PD models in vivo^[^ ^46^, ^47^ ^]^ and in vitro^[^ ^48^ ^]^ may be able to reduce motor symptoms in rodents and improve neuronal function, respectively. Similarly, it was believed that the removal of “reactive” astrocytes from the injured area of the spinal cord would be sufficient for neuronal and ultimately functional restoration—which was ultimately not supported by experimental evidence.^[^ ^49^ ^]^ Moreover, transplantation of healthy astrocytes is currently investigated clinically in amyotrophic lateral sclerosis.^[^ ^50^ ^]^ This calls into question whether astrocytic network restoration should be considered more widely as a valid therapeutic target. Having an in vitro system to study astrocytic networks in health and disease could provide a useful tool in translational research so that the mechanisms responsible for healthy astrocytic signal transmission can be characterized in greater depth and could ultimately be restored in disease.

To overcome the technical problem of close association between neuronal and astrocytic networks in culture and test whether astrocytic networks are capable of independent propagation of signals between neuronal groups, we have designed a modified MEA‐based device that we called AstroMEA. AstroMEA utilizes traditional electrodes that are optimized for detecting extracellular field potentials from which neuronal spikes can be decoded; neuronal‐astrocytic co‐cultures are grown in physically separated wells where they reach electrical maturity defined in vitro by the presence of steady action potential firing and emergence of burst events. The function of the astrocytic network is studied by applying a “bridge” composed of a densely populated 3D astrocytic monoculture which links the neuronal‐astrocytic co‐cultures that are grown on MEA electrodes in separate wells. In this device, neurons were used as tools to relay astrocytic signals to an electrophysiological recording system, and we chose well‐characterized by us^[^ ^51^, ^52^ ^]^ and easy‐to‐culture human glutamatergic induced neurones (iNeurones, iNeu) which are similar in phenotype to cortical neurones. These cells offer greater batch‐to‐batch reproducibility and purity compared to primary neuronal cultures. For the astrocytic culture, primary cortical astrocytes isolated from the rat cortex were chosen as these cells were used by us previously to enable electrical maturation of iNeu^[^ ^52^ ^]^; in the current report extra steps were taken to specifically purify cortical astrocytes from the mixed glial cultures. Despite recent advances in human‐induced astrocyte generation including by us,^[^ ^53^ ^]^ characterization of these cells is still in progress; at the same time primary human astrocytes are scarce, offer a higher batch‐to‐batch variability, and are sometimes not well characterized in terms of their brain region of origin, developmental stage, or pathological condition of the donor, thus we chose the most established cortical astrocyte culture of rat origin despite the species difference. The distance between the physically isolated groups of neuronal‐astrocytic co‐cultures was ≈8 mm, which was based on the layout of the standard six‐well MEA electrodes compatible with the Intan recording system.

We hypothesized that if an astrocytic network alone can transmit signals capable of changing neuronal firing, then electrical stimuli applied to one of the neuronal‐astrocytic co‐cultures would result in changes in neuronal firing detected at the isolated neuronal‐astrocytic co‐culture bridged by astrocytes (modeling, in a simplified fashion, isolated functional nuclei in the brain). Our hypothesis was agnostic to the direction of the change (e.g., increase or decrease) and time of the signal transfer since this was the first feasibility study of the AstroMEA system. We chose the electrical stimulation parameters for this study based on the previous literature report^[^ ^54^ ^]^ indicating that a theta‐burst of high‐frequency electrical stimulation comprised of five pulses of 100 Hz repeated ten times with 200 ms intervals with total 50 pulses lasting for 2 s per episode was able to trigger synaptically‐driven astrocytic calcium events in brain slices which contributed to the induction of long‐term plasticity. Given the known importance of astrocytic calcium events outlined above, this provided a useful starting point in our experimental setup, even though our hypothesis was agnostic to the potential contribution of non‐calcium‐related signal transmission in astrocytic networks such as direct electrical coupling via gap junctions in addition to the calcium waves.

In the current manuscript, we are presenting the results of the feasibility study using the AstroMEA that have validated the ability of our AstroMEA design to successfully separate neuronal and astrocytic processes, and its ability to detect changes in neuronal signaling following stimulation of a separate neuronal group connected via an astrocytic network. These results support our hypothesis that astrocytic networks may represent a parallel mechanism for electrical information integration and transfer, which opens up opportunities for studying astrocytic network effects on neuronal signaling under physiological and pathological conditions and offers a new conceptual perspective on the computational abilities of the brain.

## 2 Results

### 2.1 Mature Rat Astrocytes Form Gap Junctionally Coupled Networks that are Capable of Calcium Communication within 5 Days Post‐Replating In Vitro

**Figure 2:**
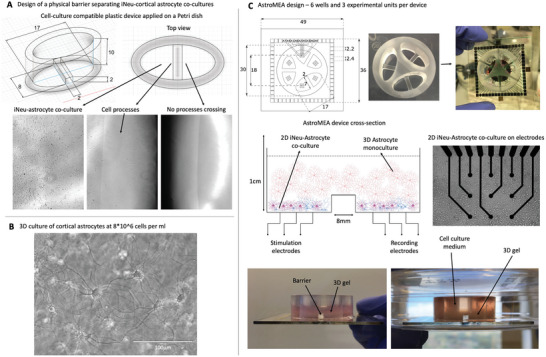
AstroMEA device design. A) A physical barrier stops axonal outgrowth into a neighboring well during 4 weeks of culture. Wells are composed of PMMA. Images taken in live cultures using a brightfield microscope (EVOS), *n* = 6. B) Astrocytes establish process contact in a 3D matrix as early as 1 day post‐plating. Images taken in live cultures using a brightfield microscope (EVOS); scale bar: 100 µm, *n* = 3. C) AstroMEA was designed by positioning the PMMA overlay onto an extracellular multi‐electrode array (MEA) with 60 electrodes, nine electrodes per well, creating three independent experimental units per six‐well MEA. 2D astrocyte‐iNeuron (iNeu) co‐cultures were grown within the wells with the axonal processes separated by a physical barrier. 3D gel containing an astrocyte network was then positioned on top of the 2D co‐cultures so as to bridge the neighboring wells.

Our first objective was to create a system that would separate neuronal and astrocytic processes in a way that would create two groups of neuron‐astrocyte co‐cultures bridged via a network of astrocytes so that our hypothesis on signal transfer within astrocytic networks can be tested. Primary rat cortical astrocytes (>90% purity, Figure S1A, Supporting Information) matured for 1 month post‐isolation from newborn rat pups and replated no more than once plus induced human neurons (iNeurons, iNeu) were used. We found that a physical barrier that is at least 2 mm high and 2 mm wide was most efficient at stopping axonal overgrowth and separating neuronal groups (**Figure** **2**A, *n* = 6 over 4 weeks); other methods including chondroitin sulfate proteoglycan coating barrier and polytetrafluoroethylene tape barrier did not reliably prevent axonal outgrowth (data not shown). In order to bridge the physically separated wells containing neuronal‐astrocytic co‐cultures, a previously published protocol for the astrocyte‐optimized 3D culture was employed.^[^ ^55^ ^]^ We observed that astrocytes were forming physical connections visible in a 3D scaffold when assessed via brightfield microscopy (Figure 2B) when seeded at a density of 5–8 million astrocytes per 1 mL of the 3D gel, which was chosen for further experiments since we hypothesized that close contacts between astrocytic processes would be necessary for gap junctional coupling that would contribute to the transmission of calcium waves and electrical coupling. We then designed a cell culture‐compatible acrylic overlay that would fit on top of a six‐well MEA, thus creating three independent experimental devices per single six‐well MEA template with nine electrodes in each of the six wells (Figure 2C).

**Figure 3:**
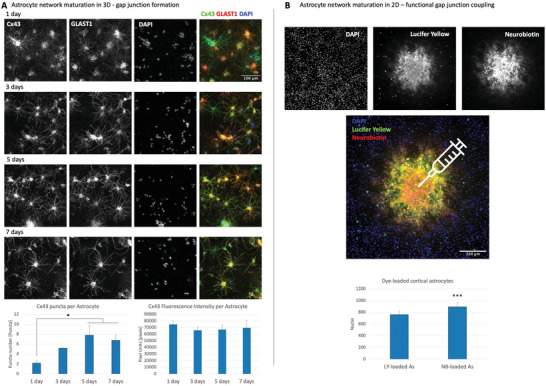
Validation of the in vitro astrocytic network maturation. A) Cx43‐containing gap junctional puncta formation and GLAST1 expression were monitored over the time course of 7 days. Gap junctional puncta, but not total connexin43 (Cx43) fluorescence, peaked around day 5 in 3D culture. Scale bar: 100 µm, *n* = 3 per timepoint. Error bars: SEM. *p*‐Values were calculated using one‐way ANOVA with Tukey post hoc test. *p < 0.05. B) Functional gap junctional coupling between cultured astrocytes after 5–7 days. Two dyes, Lucifer Yellow (457.24 Da) and Neurobiotin (322.8 Da) were injected simultaneously to visualize astrocytic syncytium. Scale bar: 250 µm, *n* = 20 injections, cells from at least 3 independent cultures. Error bars: SEM. P‐values were calculated using a paired *t*‐test. *p < 0.0005.

Next, we have explored whether our cultured astrocytes form a functional syncytium that is capable of transmitting calcium waves as well as other potential signals. Connexin43 (Cx43) is a major gap junctional protein in astrocytes^[^ ^56^ ^]^ that mediates complex forms of cell‐to‐cell communication, allowing for transmission of second messengers (including calcium and inositol triphosphate) and nutrients such as glucose and lactate between cells as well as promoting electrical coupling,^[^ ^57^ ^]^ and its deletion in the hippocampus in vivo was found to impair memory formation,^[^ ^36^ ^]^ making this protein an important candidate for mediating astrocytic signal transmission that may influence neuronal signaling. Cx43 protein pathway is complex as the protein can exist in several states including gap junctions linking astrocytes in a network, hemichannels that can open directly to the extracellular space,^[^ ^58^ ^]^ and intracellular Cx43 that may have non‐junctional functions, for example, in mitochondria.^[^ ^59^ ^]^ Under physiological conditions, gap junctions appear to be the predominant location of Cx43 while hemichannel opening increases with cell stress and inflammation^[^ ^60^, ^61^ ^]^; these gap junctional structures can be visualized microscopically as they segregate in a distinct punctate manner at cellular process contacts as opposed to the diffuse membrane or intracellular staining^[^ ^62^, ^63^, ^64^ ^]^ (limitations discussed below). We assessed the development of the Cx43‐immunoreactive puncta in 3D astrocytic cultures to estimate the timepoint at which the gap junctional network is likely to be sufficiently mature and functional; glutamate‐aspartate transporter 1 (GLAST1) counterstaining was used as an example of a predominantly astrocyte‐expressed protein^[^ ^65^ ^]^ that does not develop punctate staining, thus corroborating our observation that Cx43 puncta formation was a biological phenomenon and not an artifact due to the process fragmentation following cell stress (glial fibrillary acidic protein (GFAP), a common astrocyte lineage marker, was not used as we noted that anti‐GFAP antibodies performed poorly in our 3D staining compared to anti‐GLAST). We found that although physical cell process contacts start occurring after 1 day in 3D culture and diffuse Cx43 expression was evident at that point, gap junctional puncta did not become distinct until day 5 in gel (**Figure** **3**A). This staining showed that the total expression of Cx43 did not change significantly over time, while the number of puncta increased during the first 5 days, after which it reached a plateau. This result allowed us to choose 5–7 days as the minimal time period for 3D astrocyte culture to bridge isolated neuronal groups; longer astrocyte bridge culture periods were avoided for the purposes of AstroMEA as it was noted that the neuronal projections start growing abundantly into the 3D matrix after 2 weeks (data not shown). Astrocytes remained viable in 3D cultures for at least 3 months.

Cx43 staining has important limitations, including the fact that Cx43‐containing gap junctions can exist in a closed state, or become internalized in annular junctions which are not functional.^[^ ^58^ ^]^ To confirm the functional maturation of gap junctions in an astrocyte syncytium, we studied dye spread patterns following a microinjection of a single cell in 2D astrocyte cultures after 5–7 days post‐replating (3D gel was not used due to technical difficulties around injection and analysis) and found that astrocytes formed vast networks capable of transferring Neurobiotin (NB) and Lucifer Yellow (LY) dyes (Figure 3B). LY has a molecular weight of 457.24 Da that passes through Cx43‐containing gap junctions but is too large for some other junctional channels such as those composed of Cx30 and Cx45,^[^ ^66^, ^67^ ^]^ while NB is a smaller molecule of 322.8 Da that can pass through a wider range of junctional channels. As expected, the number of NB‐labeled cells typically exceeded the number of LY‐labeled cells (Figure 3B), suggesting that several members of the connexin and pannexin family beyond connexin43 may contribute to the functional coupling in cultured cortical astrocytes; this was also corroborated by our mRNA sequencing data (not shown).

Next, we investigated the ability of cultured astrocytes to communicate via calcium waves due to the importance of this mode of communication in multiple astrocytic functions. The timepoints of gap junctional emergence estimated above were chosen for calcium wave experiments given the roles of Cx43 in calcium wave propagation,^[^ ^68^ ^]^ although non‐Cx43‐junctional calcium wave spread was also described.^[^ ^69^, ^70^ ^]^ In the AstroMEA setup, astrocytes can be exposed to two main types of potential calcium wave triggers—chemical transmitter release (glutamate, ATP) from neurons, and electrical stimulus itself as well as neuronal electrical signals.

**Figure 4:**
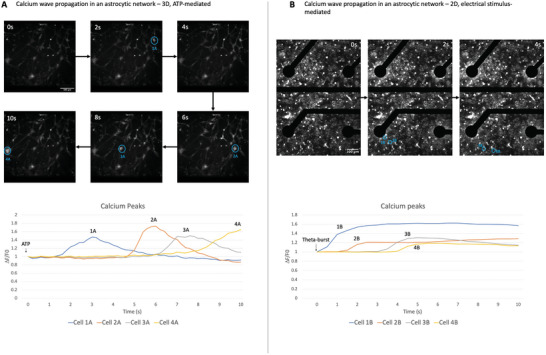
Calcium wave propagation in cultured astrocytes. A) A large calcium wave was induced in a 5‐day matured 3D astrocytic culture by application of 100 µM ATP. Directionality of the wave propagation could be visualized by sequential cell body activation. Calcium elevations were measured with Fluo‐4 non‐ratiometric dye. Scale bar: 100 µm, *n* = 3. B) Calcium waves were induced in a 5‐day matured 2D astrocytic culture by an electrical theta‐burst charge‐balanced biphasic current stimulation that consisted of five pulses of 100 Hz stimulation repeated ten times with 200 ms intervals. Scale bar: 100 µm, *n* = 3.

To test the well‐described ability of astrocytic networks to respond to chemical stimuli such as ATP^[^ ^71^ ^]^ in our system, we induced live calcium waves via acute application of 100 µm ATP in 5‐day‐old 3D cultures. Calcium elevations in cell bodies and large processes were visualized with a non‐ratiometric calcium dye Fluo‐4, which allowed us to clearly detect calcium propagation between cell bodies through astrocytic processes (**Figure** **4**A; Video S1, Supporting Information). Interestingly, calcium wave spread was detected in several directions including the direction opposite to the ATP diffusion gradient, suggesting an astrocyte‐to‐astrocyte signal transmission as opposed to individual cell activation due to diffusional ATP spread (Video S2, Supporting Information). This technique is not sensitive enough to capture the whole complexity of calcium signaling (e.g., events at small processes), but it allowed us to confirm that astrocytes form a functional communicating network in a 3D scaffold.

Astrocytes have also been shown to elevate intracellular calcium to response to electrical stimuli.^[^ ^70^ ^]^ To test whether astrocytes can respond to electrical theta‐bursts directly, we cultured 2D astrocytes on MEAs for 5 days and employed the stimulation paradigm that was previously suggested to induce astrocytic calcium transients in a synaptically dependent manner^[^ ^54^ ^]^ using an Intan stimulation system. We could not use 3D cultures in this setup due to technical limitations as our MEAs were made of thicker glass than optimal for calcium imaging, and imaging as well as calcium dye loading in a layer of 3D gel attached to the thick glass was not feasible. We found that astrocytic monocultures were able to respond to electrical stimulation with a calcium wave radiating from the active electrodes (Figure 4B, Video S2, Supporting Information), and these cytoplasmic calcium elevations were sustained for at least 10 s. Thus, astrocytes can generate calcium responses to theta‐bursts directly as well as in response to chemical transmitters that may become released from activated neurons. The speed of calcium wave propagation among cell bodies and large processes was ≈40–70 µm s^−1^.

### 2.2 Astrocytic Network Propagates Signals between Neuron‐Astrocyte Co‐Cultures

We have hypothesized that if astrocytic signals can change neuronal firing, and that astrocytes can transmit signals to each other, astrocytic networks could be able to transmit signals between otherwise electrically independent groups of neurons. An extracellular electrode array could be employed to detect electrical field potential changes associated with altered firing of neuronal cultures.

**Figure 5:**
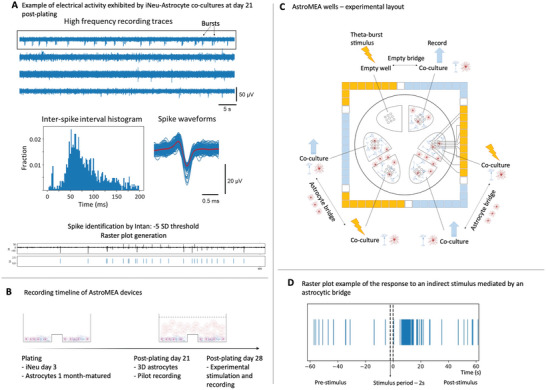
Electrophysiological analysis of AstroMEAs. A) iNeuron (iNeu)‐astrocyte co‐cultures electrically mature on MEAs over 21 days. Presence of typical spike waveforms and burst patterns indicated electrical maturation of the network. B) Recording timeline of AstroMEA devices. A 3‐week period of electrical maturation of 2D co‐cultures was followed by a 1‐week period of additional 3D astrocytic culture maturation on top to bridge the neighboring wells. C) Experimental layout of the AstroMEA wells. Four independent devices (each representing a biological replicate for the astrocytes) with the indicated layout were used for recordings; each well contains nine electrodes. D) Raster plot example showing the detection and classification of pre‐ and post‐stimulation signaling patterns on AstroMEAs. The period of stimulation (2 s plus 2 ms directly following the stimulus) was excluded from the analysis both in stimulated and non‐stimulated wells due to the presence of electrical artifacts.

According to the initial characterization experiments, iNeu‐astrocyte co‐cultures developed electrical activity consistently detectable by MEAs after 2–3 weeks post‐co‐plating and continued maturing with more complex signaling patterns such as bursts developing after around 3 weeks in co‐culture (**Figure** **5**A; Figure S1B, Supporting Information). In the absence of astrocytes, electrical maturation of iNeu was slower (Figure S1B, Supporting Information). Therefore, we chose to mature iNeu‐astrocyte co‐cultures in 2D for 3 weeks on AstroMEA electrodes, then apply an astrocyte monoculture in 3D for 1 week, followed by the final recording (Figure 5B). Prior to the 3D culture application, electrical activity of iNeu‐astrocyte co‐culture was confirmed, and non‐viable co‐cultures were excluded from further analysis.

In order to detect the indirect signal transmission between the iNeu‐astrocyte co‐culture groups, one of the groups was stimulated with a theta‐burst.^[^ ^54^ ^]^ Although another group of iNeu‐astrocytes was not stimulated directly, the stimulation period plus 2 ms after the stimulus ended was excluded from the recording analysis in all electrodes since electrical artifacts occurred in the entire system during stimulation. Up to five stimulation episodes spaced 10 min apart were included per recording at a single device, and four devices in total (each representing a biological replicate for the astrocytes, i.e., astrocytes from a different rat pup batch) with the layout presented in Figure 5C were used. No devices matching our experimental quality controls (presence of spikes at the baseline in iNeu‐containing wells and 3D astrocytic bridges covering the barriers fully) were excluded from the analysis. Intan hardware was used for stimulation and recording, and Intan software was used to automatically extract spikes from the recorded traces using a threshold calculated at −5 standard deviations (from the baseline noise) for each electrode (Figure 5B). This allowed us to compare the baseline level of signaling at a particular electrode with the signaling pattern following the theta‐burst, both in directly stimulated co‐cultures and in co‐cultures stimulated indirectly via an astrocytic bridge (Figure 5D). Empty wells with no cells were used as a control condition for the effects of the direct stimulation on the electrodes, and a co‐culture bridged with an empty stimulated well via an empty 3D gel bridge was used as a control condition to eliminate the possibility of the electrical stimulus traveling via the 3D matrix and/or cell culture medium (Figure 5C). A single stimulated (directly or indirectly) electrode acted as an experimental unit; electrodes with unstable widebands and impedance values over 100 kΩ were excluded from the analysis as potentially faulty.

**Figure 6:**
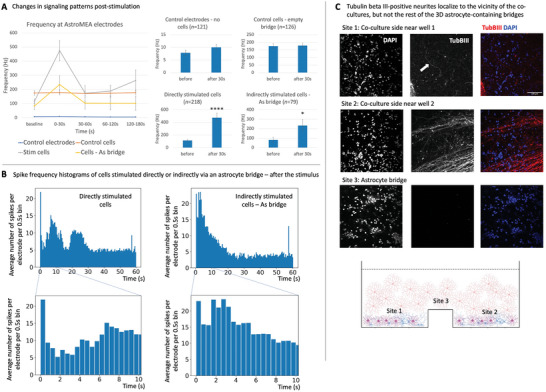
Signal transmission via astrocytic networks. A) Changes in signaling patterns after a theta‐burst stimulation. iNeuron (iNeu)‐astrocyte (As) co‐cultures that were directly stimulated, and those which were stimulated indirectly via an astrocytic bridge, showed significantly increased action potential firing patterns within 30 s directly following the stimulus compared to the baseline firing rate. Changes in spike frequency were analyzed with a paired t‐test. Error bars: SEM. *p*‐Values were calculated using a paired *t*‐test. *p < 0.05, ****p < 0.00005. B) Spike frequency histograms of directly and indirectly stimulated cells directly following the theta burst. Each bin is 0.5 s wide and represents an average number of spikes per electrode for all measured electrodes in a given condition. C) AstroMEA device faithfully separated neuronal groups. Absence of axonal projections bridging the neighboring wells was confirmed using anti‐tubulinβIII staining. Scale bar: 100 µm, *n* = 8.

To quantitatively evaluate the effects of the theta‐burst stimulation, first, the “baseline” firing frequency was established for each stimulation event by measuring the spikes at each electrode during the 5 min interval directly prior to the theta‐burst. Next, after exclusion of the 2 s stimulation interval plus 2 ms directly following it, frequencies at the same electrode were measured at 0–30 s, 30–60 s, 60–120 s, and 120–180 s post‐stimulation (**Figure** **6**A). This provided us with an overview of the post‐stimulation signaling patterns, where the first 30 s after the theta‐burst showed the most pronounced firing frequency increase in the directly‐stimulated cells, and also in the indirectly stimulated cells connected to the directly stimulated cells via an astrocyte bridge. It was noted that the directly stimulated cells sometimes exhibited elevated firing frequencies for several minutes after the stimulus while the indirectly stimulated cells with an astrocyte bridge returned to the baseline firing rate quicker. Empty electrodes did not show profound artifacts in response to the direct theta‐burst as expected, and neither did the indirectly stimulated cells bridged to the stimulated empty electrodes via an empty 3D matrix bridge (Figure 6A). We observed a degree of variability in spike detection sensitivity between MEA devices and individual MEA wells at baseline (pre‐stimulation), which could be related to the MEA production differences and the co‐culture arrangement on the electrodes. For instance, electrodes surrounded by a tight group of cells could pick up more signals than sparsely populated electrodes; for this reason, we also could not faithfully estimate whether the transmitted signal frequency was preserved by an astrocyte bridge, e.g., whether the individual neurons in the indirectly stimulated well increased their firing rate by 100 Hz since the MEA electrodes record from a group of cells (25 000 iNeu and 25 000 astrocytes were plated per well on top of 9 electrodes) which can have overlapping signaling patterns. We additionally noted that the spike events were generally smaller in amplitude in the wells with an astrocyte bridge, likely due to the higher rate of metabolism due to a much greater number of cells per well (≈0.5 mL of the gel per well at ≈8 × 10^6^ astrocytes mL^−1^, giving rise to ≈4 100 000 cells in the wells with astrocytic bridges compared with 50 000 cells in the wells without) that resulted in lower pH. Devices were fed daily to compensate for the high rate of medium consumption, and the culture medium was exchanged around 30 min prior to commencing the AstroMEA stimulation and recording in all conditions. An additional factor that could contribute to the decreased spike amplitude in astrocyte‐bridged wells was a slight contraction and movement of the gel with cells over several days, leading to the lifting of the cell layer away from the electrodes.

Given the greatest observed differences in firing in the directly as well as indirectly stimulated cells within the first 30 s post‐theta‐burst (Figure 6A) and also as noted after examining raster plots (Figure 5D), we have focused the statistical analysis on the differences between the baseline and the following 0–30 s post‐stimulus interval using a paired t‐test. The test revealed a very significant increase in the post‐stimulus firing frequency in the directly stimulated cells (p = 3.869e‐7, *n* = 218 stimulated electrodes), and a small significant increase in the post‐stimulus firing frequency in indirectly stimulated cells bridged to the directly stimulated cells via an astrocyte bridge (*p* = 0.007643, *n* = 79). We found that in each bridged culture, only a small to medium proportion of electrodes in the indirectly stimulated well detected a change, but all iNeu‐astrocyte co‐cultures that were stimulated indirectly via an astrocyte bridge visibly responded to the stimulus in at least one electrode. Directly stimulated empty electrodes (*p* = 0.1349, *n* = 121) and control cells indirectly stimulated via an empty 3D matrix (*p* = 0.3628, *n* = 126) did not exhibit significant changes in detected signals (Figure 6A).

We have further tested whether the presence of neurons in the directly‐stimulated well was necessary for the signal initiation in astrocytes, or would the direct electrical stimulation of astrocytes be sufficient to initiate the response as suggested by the results shown in Figure 4B. We thus modified the AstroMEA layout and included a pure astrocyte culture in the well for direct stimulation, and the iNeu‐astrocyte co‐culture in the bridged well served as an indicator of the signal propagation through the astrocytic bridge as before (Figure S2, Supporting Information). This pilot study indicated that the direct electrical stimulation of astrocytes in the absence of neurons was able to initiate some response in the bridged cells in the form of elevated neuronal firing (*p* = 0.7646, *n* = 12). This response did not reach significance and was smaller proportionally (>150% baseline) compared to the response when iNeu were present in the directly stimulated well (>280% baseline), albeit this must be interpreted with caution due to the low *n* in the pilot study. Overall, it suggests that a direct electrical stimulus might be sufficient to cause some astrocytic signal initiation, but excitatory neuronal activity may further enhance such signal potentially due to, e.g., chemical transmitter release. Curiously, we noted a short‐lived (up to 30 s) elevated “spike‐like” activity with small amplitudes in directly electrically stimulated astrocytes (Figure S2, Supporting Information), which might relate to the similar phenomena previously described in vitro by others.^[^ ^72^ ^]^

In order to further analyze the speed of the stimulus transmission, we have generated spike frequency histograms for the directly and indirectly stimulated cells bridged via astrocytic networks where the bin size was 0.5 s, each bin showed the number of spikes per stimulated electrode averaged for all electrodes from the relevant condition (Figure 6B). The histograms showed that the cells stimulated indirectly via an astrocytic bridge often exhibited a signaling peak a few milliseconds after the stimulus end and also ≈2–3 s after the stimulus end, after which the probability of increased signaling steadily declined over 20 s (Figure 6B). This suggests that the astrocytic signal transmission that was able to change neuronal signaling pattern could take from 0.5 to 3 s to travel through approximately 8 mm bridge distance, thus traveling at the speed of 2.7–16 mm s^−1^ (assuming that the response time is measured from the end of the theta‐burst stimulus, although it is possible that the astrocytic signal is initiated prior to the completion of the theta‐burst, making speed estimates closer to 1.6–3.2 mm s^−1^). This is faster than the previously reported speed range of calcium wave spread in cultured rodent astrocytes of ≈8.6 µm s^−1^,^[^ ^42^ ^]^ and is faster than the large calcium wave spread which involved cell bodies observed in astrocytes by us in response to ATP or a theta‐burst of ≈40–70 µm s^−1^ (Figure 4), although a rapid onset of astrocytic calcium responses to sensory stimulation and cortical circuit activity comparable to our result has been previously reported in vivo.^[^ ^25^, ^39^ ^]^ It is possible that finer calcium events involving astrocytic processes which we could not faithfully capture with our calcium dyes, or non‐calcium transfer mechanisms such as electrical coupling, could have contributed to the astrocytic signal transfer that could rapidly alter neuronal firing.

The cells that were stimulated by a theta‐burst directly also exhibited a pronounced immediate peak directly after the stimulation end as well as several waves of increased firing ≈7 and 20 s post‐stimulus. Interestingly, events that appeared as reverse, or antidromic, signal propagation were occasionally observed where bursts of activity in the indirectly stimulated cells bridged via astrocytic networks might have correlated with a slightly delayed activity in the bridged well which was stimulated originally; however we did not quantify these events further since it was not possible to separate the possible delayed effects of the direct stimulation from the potential antidromic astrocytic signal transmission.

### 2.3 AstroMEA Reliably Separates Astrocytic and Neuronal Processes

Finally, we conducted post‐recording quality control checks on the PFA‐fixed astrocytic bridges used in the AstroMEA experiments to ensure that neuronal processes did not bridge the iNeu‐astrocyte groups in the experimental wells. We stained the bridges against a neuronal marker tubulinβIII and found neuronal processes gently ingrowing into the 3D matrix around the 2D co‐culture sites in the wells (Figure 6C)—this pattern was expected based on our preliminary experiments (data not shown). No neuronal staining was found in the astrocytic bridge area where the separating barrier was positioned, while abundant non‐neuronal nuclei indicated the presence of an astrocytic network (Figure 6C). This corroborated the interpretation of our data suggesting that the astrocytic bridge acted to transmit the signal which changed signaling patterns in separated neuron‐astrocyte groups.

## 3 Discussion

Astrocytic signaling, which is typically associated with calcium wave transfer, has been proposed to have important roles in normal brain physiology, and its dysregulation is often found in association with disease. For example, astrocytic calcium elevations and gap junctional coupling were found to be necessary for long‐term potentiation induction in hippocampal neurons.^[^ ^22^, ^36^ ^]^ At the same time calcium abnormalities in astrocytes were found in association with alpha‐synuclein lesions^[^ ^73^, ^74^ ^]^ and PD‐associated mutation LRRK2,^[^ ^75^ ^]^ Huntington's disease‐associated mutation,^[^ ^76^ ^]^ and inflammation^[^ ^61^, ^77^ ^]^—diseased astrocytes have a tendency to show a reduced calcium elevation response to physiological stimuli but a trend toward longer‐term elevated calcium levels; abnormally elevated calcium may further perpetuate mitochondrial dysfunction,^[^ ^78^ ^]^ alpha‐synuclein aggregation,^[^ ^79^ ^]^ and ultimately cell death.^[^ ^80^ ^]^ Gap junctional coupling is an important underlying mechanism regulating astrocytic calcium signaling; in disease states, gap junctions have a tendency to close while hemichannels open,^[^ ^60^, ^61^, ^81^ ^]^ which can contribute to the explanation of the chronic calcium level elevations in astrocytes coupled with a decreased ability to propagate calcium waves in response to stimuli. In accordance with this, pathological ischemic conditions downregulate transjunctional potassium‐mediated electrical coupling between astrocytes in brain slices^[^ ^82^ ^]^; this ability to propagate electrical currents may represent another important mechanism of signal transmission between astrocytes in addition to the calcium wave transfer. Despite the increasing evidence of the importance of astrocytic signaling, measuring the effects of astrocytic signal transmission on neuronal firing is challenging since traditional astrocyte‐neuron co‐cultures or brain slice cultures do not allow for differentiation between an astrocyte‐to‐neuron‐to‐astrocyte signal transmission and a signal propagation within a network composed of a single cell type.

Conceptually, the ability of astrocytes to encode and transmit signals in parallel to neuronal firing has important implications for our understanding of the computational properties of the brain. The “astrocentric hypothesis” of brain function has been originally proposed several decades ago;^[^ ^43^ ^]^ it suggested that astrocytic coupling is essential for cognition and consciousness.^[^ ^44^ ^]^ Curious links exist to support this view. For instance, it has been noted that increased astrocyte‐to‐neuron ratio, highly ramified (hence potentially able to create more connections) astrocytic morphology, and increased variety of astrocyte subtypes correlate with behavioral and cognitive complexity of organisms to a greater extent than an increase in neuronal complexity—human cortical protoplasmic astrocytes present with a staggering 27‐fold increase in volume, 2.55‐fold increase in diameter, ten times more processes, over 20‐fold increase in the number of interacting synapses, and over five times faster calcium waves compared to their rodent counterparts, while human neurons are only ≈2.5 times larger.^[^ ^42^, ^83^ ^]^ While neuronal connectivity and signaling were traditionally considered to be the foundation of the computational ability of the brain, it has been recently demonstrated mathematically that astrocytic networks are capable of reaching a comparable computational complexity,^[^ ^84^ ^]^ and inclusion of “digital astrocytes” in a neural net machine learning algorithm was found to improve the model's computational performance.^[^ ^85^ ^]^ Interestingly, the brain of the famous physicist Albert Einstein contained lower neuron‐to‐glia (non‐neuronal cells including astrocytes) ratio in a brain area associated with mathematical performance,^[^ ^86^ ^]^ and astrocytic processes in his brain were distinguished by a higher complexity compared to an average individual—although causal relationship between astrocytic morphologies and cognitive performance have been disputed.^[^ ^87^ ^]^ It is also noteworthy that in the healthy rodent brain, striatal astrocytes can exhibit numerous spontaneous calcium signals that are not influenced by the neuronal input from the cortex,^[^ ^76^ ^]^ and that astrocytic calcium transients in the spinal organotypic cultures are tetrodotoxin (TTX)‐resistant (TTX blocks neuronal activity),^[^ ^61^ ^]^ suggesting that although astrocytes can respond to the local neuronal signals with calcium waves under specific circumstances,^[^ ^54^ ^]^ they may also offer an additional computational layer that is parallel to neurons which operates at its own timescales. Similar observations were made in rodent brain slices, where 95.4% of spontaneously‐active neurons correlated with a spontaneously‐active astrocyte, but only 61% of spontaneously‐active astrocytes correlated with a spontaneously‐active neuron.^[^ ^88^ ^]^ This is additionally supported by the experiments where reduction in the hippocampal astrocytic communication was casually linked to decreased learning abilities in rodents.^[^ ^27^, ^36^ ^]^ Accordingly, reduction in gap junctional coupling was found in association with general anesthetics, further linking this pathway to consciousness and cognition.^[^ ^89^ ^]^

However, the practical implications of the astrocentric hypothesis turned out to be challenging to test experimentally or to find translational applications of this paradigm shift away from neuronal‐exclusive information processing. For instance, even though profound changes in the astrocytic gene and protein expression as well as function were widely described in human brain conditions ranging from demyelination^[^ ^90^, ^91^ ^]^ to autism spectrum^[^ ^92^, ^93^, ^94^ ^]^ and dementia,^[^ ^95^ ^]^ it always left a question of whether the functional symptoms experienced by individuals were ultimately the result of the altered neuronal function due to the diminished metabolic support or release of toxic substances by astrocytes.

To address this long‐standing question, we have designed and functionally validated a device called AstroMEA which utilized electrical properties of neurons to detect signal propagation via an astrocytic network that can change neuronal signaling. Such astrocytic signals were rapid, reaching speeds of several mm per second, and could potentially be mediated by fast calcium events^[^ ^25^, ^31^ ^]^ and/or gap junctional electrical current transfer.^[^ ^82^ ^]^ Interestingly, high‐frequency voltage oscillations have been described in astrocytes in vitro that may be related to some extent to junctional coupling and calcium events,^[^ ^72^ ^]^ which highlights further possibilities to deepen our knowledge of methods of astrocytic communication in the future.

It was noted that the astrocytic networks did not transfer the signal in a totally linear fashion; for instance, there was a clear difference in the post‐stimulation signaling patterns in iNeu‐astrocyte co‐cultures stimulated directly or indirectly via an astrocyte bridge (Figure 6). While direct electrical stimulation produced a more long‐lasting excitatory response in the cell firing, the effect of the indirect stimulation was shorter‐lived. This suggests that astrocytic networks may *integrate* ^[^ ^96^ ^]^ and *transmute* electrical and chemical signals from distant signaling hubs as opposed to purely transferring them faithfully. In particular, it is possible based on the data observed that a high threshold of stimulation would be needed to activate this rapid astrocytic signal transfer since individual spontaneous action potential bursts or single electrical pulses (data not shown) did not produce the responses in the bridged wells – albeit this could also represent a limitation of the sensitivity of our technology. Such a mechanism would correspond well with the “astrocentric hypothesis” view of consciousness^[^ ^43^ ^]^ where astrocytic networks may integrate strong signals arising from various sensory stimuli. It is possible that a certain frequency and amplitude of electrical signals, especially if accompanied by abundant chemical transmitter messages, need to co‐occur to enable the initiation of the astrocytic network signal. Thus, astrocytic and neuronal nets may have "non‐redundant functions" in long‐range information transfer and processing in the brain where neurones could serve to transmit the signals rapidly and faithfully as to enable rapid responses with their fast, all‐or‐nothing signals, while astrocytes transform the signal by helping to sieve out the noise and respond to the strongest, potentially most important stimuli. Such a view could explain how astrocytic networks could help specific memory allocation while random neuronal overactivation had an opposite effect,^[^ ^27^ ^]^ and this mechanism may go wrong in disease such as Alzheimer's where astrocytic signals become overactive and no longer coordinated with neuronal inputs.^[^ ^38^ ^]^

Although seemingly small, a conceptual shift towards regarding astrocytic network information processing as valuable in its own right opens up interesting translational possibilities. Given that the abnormal calcium signals, as well as dysregulated expression and function, of proteins linking astrocytes in a syncytium and/or allowing for the release of chemical transmitters such as connexins, pannexins, and aquaporins are involved in brain disorders such as Parkinson's,^[^ ^75^, ^97^ ^]^ Alzheimer's,^[^ ^98^, ^99^ ^]^ epilepsy,^[^ ^35^, ^100^, ^101^ ^]^ amyotrophic lateral sclerosis,^[^ ^102^, ^103^ ^]^ multiple sclerosis,^[^ ^81^ ^]^ Down's syndrome,^[^ ^104^ ^]^ and more, AstroMEA could provide an in vitro translational testing platform differentiating between astrocytic and neuronal network functions for the first time. For instance, disease‐associated mutations may be introduced in the bridging astrocytes to study how these may influence signal propagation, or small molecules targeting astrocytes could be tested, albeit their diffusion through a 3D matrix needs to be confirmed. It is also possible to reverse the cell arrangement in the device and introduce neurons in the 3D bridge so that the effects of mutations in neuronal versus astrocytic networks may be compared directly.

## 4 Limitations of the Study

There is a number of limitations of the AstroMEA study presented here that have to be considered. Firstly, astrocytic and neuronal networks do not exist in isolation under physiological conditions (with the exception of neuronal fibers in the peripheral nervous system), and our data (Figure S1B, Supporting Information) suggest that neuronal cultures exhibit a delay in electrical maturation in the absence of astrocytes. This may also apply the other way round, with astrocytic networks lacking in maturation and full range of physiological function when devoid of neuronal cell types, which is supported by the published research showing that neuronal surface proteins could enhance astrocytic morphological complexity,^[^ ^105^ ^]^ and that the presence of neurons promoted astrocytic connexin expression and network maturation independent of the neuronal electrical activity.^[^ ^106^ ^]^ Thus, astrocytes and neurons may provide mutual trophic support. The addition of pre‐fixed, non‐functional neurons into the astrocytic bridge could provide the structural support to promote astrocytic network maturation.^[^ ^105^ ^]^ Second, roles of other brain cell types such as oligodendrocytes and microglia were not considered in the AstroMEA design but could be incorporated in future experiments. For example, activated microglia can release cytokines that decrease astrocytic coupling via gap junctions while opening hemichannels,^[^ ^107^, ^108^ ^]^ thus dysregulating calcium signaling, which is relevant to inflammatory disease models. Thirdly, our knowledge of the exact mechanisms that trigger signal initiation and propagation by astrocytes can be deepened. For example, our data suggested that a direct stimulation of astrocytes may be able to trigger some astrocytic signal propagation, but it is also likely that the presence of neurons further adds to the intensity of the signal. In future experiments, direct application of chemical transmitters such as glutamate to selected parts of the 3D astrocyte‐containing gel and comparison of the AstroMEA response with the direct electrical stimuli could shed further light on the roles of electrical versus chemical stimuli in the initiation of astrocytic signals. Likewise, it is not yet clear if gap junctional coupling and/or chemical transmission via receptor engagement is key for astrocytic network communication, and to what extent. Specific peptide blockers of Cx43‐containing gap junctions + hemichannels (there are no reliable gap junction‐exclusive blockers) such as GAP27 and GAP26^[^ ^109^ ^]^ can be used to address this question, although the ability of these peptides to penetrate through the 3D gel matrix needs to be tested. Carbenoxolone, a non‐specific small molecule gap junctional blocker, would not be recommended for this experiment since its off‐target effects, including those on neurons, have been reported.^[^ ^110^ ^]^ Alternatively, astrocytes can be plated sparsely throughout the 3D matrix, leaving gaps between individual cells,^[^ ^70^ ^]^ although extensive further optimization to ensure the lack of direct contacts would be required. Knock‐out strategies targeting different connexin/pannexin family members as well as their combinations^[^ ^111^ ^]^ would be the best tools to study the roles of gap junctional communication in astrocytic network signal transfer. Specific receptor blockers and/or knock‐out lines can help delineate which chemical transmitters (such as ATP or glutamate) play important roles in inter‐astrocytic signaling. Similarly, it has not been determined whether calcium signals or other mechanisms such as electrical coupling enable the rapid signal transfer within astrocytic networks. Calcium dye loading that was used in the current study allowed us to visualize calcium events at the cell bodies and large processes, which did not correspond in speed to the signal propagation detected at the AstroMEAs, suggesting that more sensitive calcium detection techniques such as GCaMPs capable of detecting calcium events at fine processes may shed further light on this question. Further, regional differences between astrocytes from different parts of the brain may play a role in determining their network properties,^[^ ^112^, ^113^, ^114^, ^115^ ^]^ while only cortical astrocytes were tested in our setup. For instance, it is possible that just as different neuronal subtypes can have opposing effects on neighboring neurons (excitatory versus inhibitory), different astrocytic subtypes could transfer unique types of signals within their networks with specific effects on neuronal, or astrocytic, signaling. It also must be noted that only glutamaterigic neurons were employed in the current study, and it could be of interest to test the effects of the astrocytic network transmission on other neuronal types since astrocytic gap junctionally‐coupled network control of inhibitory barrage firing was described in hippocampal slices.^[^ ^21^ ^]^ Finally, different electrical stimulation protocols should be tested in order to characterize the properties of the astrocytic network in more depth; this can include a lower frequency stimulation protocol (2 ms bipolar voltage pulses delivered at 17 Hz over 3–8 s) previously shown to induce calcium elevation and wave propagation in cultured astrocytes.^[^ ^70^ ^]^

## 5 Conclusion

Overall, this study provides the first evidence of the feasibility of non‐neuronal long‐distance signal transfer by astrocytic networks at rapid timescales which can independently influence neuronal signals and offers an in vitro testing platform with translational relevance.

## 6 Experimental Section

### Primary Rat Cortical Astrocyte Microdissection and Maturation

Rat pups were collected at postnatal days 1–3; brains were extracted, and cerebral cortices were microdissected. Meninges and major vessels were removed. Dissected brain regions were triturated and incubated in Digestion Medium containing papain (1.5 U mL^−1^), DNase I (60 µg mL^−1^), and l‐cysteine (240 µg mL^−1^) in MEM (all reagents from Sigma) for 30 min at 37 °C. Following the incubation, digested tissue was washed with MEM and pelleted at 300 × *g* for 10 min, supernatants discarded, and cell pellets plated in Astrocyte Maintenance Medium containing 10% heat‐inactivated FBS (Gibco) and L‐glutamine (200 mm, Sigma) in 4500 mg L^−1^ glucose DMEM (Sigma) supplemented with antibiotic‐antimycotic (Gibco) on PDL (Sigma)‐coated flasks. Cells were left to adhere for 6 days followed by full medium replacement, after which cultures were fed by half medium change every 3 days for ≈1 month. At that time, cultures were dissociated with Accutase (Gibco), counted, and re‐plated on PDL + laminin (Sigma)‐coated coverslips, Petri dishes, MEA devices, or in 3D gels. Astrocyte culture purity at this stage was estimated to exceed 90% based on GFAP and Aldh1L1 astrocytic marker expression (Figure S1A, Supporting Information).

### iNeuron‐Astrocyte Co‐Cultures

Induced neurons (iNeurons, iNeu) were generated according to previously published protocols.^[^ ^51^, ^52^ ^]^ Briefly, NGN2‐targeted stem cells were maintained in StemFlex medium (Gibco) on GelTrex (Gibco)‐coated plates. To prepare for neuronal induction, stem cells were dissociated with Accutase (Gibco) and re‐plated on GelTrex‐coated plates in ROCK inhibitor (Tocris)‐containing StemFlex. After 1 day, neuronal induction was started by medium replacement with doxycycline (1 µg mL^−1^, Sigma)‐supplemented Neuronal Induction medium containing 1% N_2_ supplement, 1% GlutaMAX, 1% non‐essential amino acid supplement, 2‐mercaptoethanol (50 µm), and antibiotic‐antimycotic in DMEM/F12 (all reagents from Gibco). Neuronal Induction medium was replaced daily for 3 days, after which the cells were dissociated with Accutase, counted, and re‐plated on PDL + laminin (Sigma)‐coated Petri dishes or AstroMEA devices in 1:1 proportion with astrocytes (25 000 cortical astrocytes + 25 000 iNeu per AstroMEA well on top of six electrodes). Co‐cultures were maintained in Neuronal Maintenance medium containing 1% GlutaMAX, 2% B27 supplement (Gibco), 2‐mercaptoethanol (50 µm), BDNF (10 ng mL^−1^, R&D systems), NT3 (10 ng mL^−1^, R&D systems), and antibiotic‐antimycotic in Neurobasal (Gibco). Neuronal Maintenance medium was supplemented with doxycycline for the first 4 days of co‐culture when the medium was replaced daily, after which doxycycline was withdrawn and co‐cultures were fed every 2 days by half medium change.

### 3D Astrocyte Culture

3D gels were optimized according to a previously published protocol.^[^ ^55^ ^]^ Briefly, cortical astrocytes matured for one month in Astrocyte Maintenance Medium as described above were dissociated in Accutase, resuspended in a pellet of high density (≈50 × 10^6^ mL^−1^), and counted. In parallel, 3D gel stock was prepared on ice by mixing the high concentration rat tail collagen I (BD) to which 10× DMEM (Sigma) and NaOH were added according to the manufacturer's instructions, growth factor‐reduced Matrigel (BD), and hyaluronic acid (HyStem Kit, Sigma) at the final concentration of 3 mg mL^−1^ collagen, 1.5 mg mL^−1^ Matrigel, and 1.5 mg mL^−1^ hyaluronic acid. Astrocyte suspension in Neuronal Maintenance medium was then added to the gel to create the final density of 8 × 10^6^ astrocytes per ml, this density was chosen empirically based on the observation of process contact within the first 1–3 days of culture; Neuronal Maintenance medium with no cells was added to the control empty gels. Liquid gel stocks were then rapidly plated in Petri dishes on coverslips or in AstroMEAs. 3D cultures were allowed to solidify at 37 °C for 30 min prior to addition of Neuronal Maintenance medium on top of the gels. Cultures were fed daily by half medium change. Approximately 50–100 µL gels were used for immunostaining and calcium imaging, and ≈500 µL gels were used in AstroMEAs to fully cover the bridges.

### 3D Culture Immunostaining

Astrocytes grown in 3D gels on coverslips or AstroMEA bridges were fixed with 4% PFA at specified timepoints (1–7 days in culture for gap junctional expression timeline cultures, and day 7 in culture for AstroMEA bridges) for 30 min at room temperature, after which the gels were extracted from the AstroMEA device if applicable, washed in PBS for 1 h and incubated in blocking and permeabilization buffer containing 5% donkey serum (Abcam) and 0.5% tritonX‐100 (Sigma) in PBS for 1 h at room temperature. Primary and secondary antibody incubations were conducted in a staining buffer containing 3% donkey serum and 0.3% tritonX‐100 for 2 days at 4 °C on a rocking shaker each with three 1 h PBS washes in between. Nuclei were visualized with DAPI (ThermoFisherScientific) added to the secondary antibody solution. Gels were kept in PBS for storage and temporary drained for imaging. During imaging, gels were re‐positioned into 35 mm µ‐dishes (Ibidi) for inverted confocal microscopy. At least three independent gels per condition were included in the analysis.

List of antibodies and dyes (all used at 1:500 final dilution):

Anti‐connexin43 rabbit – Abcam ab11370
Anti‐ GLAST1 mouse – Miltenyi Biotec 130‐095‐822
Anti‐tubulinβIII mouse – Proteintech 66240‐1‐Ig
Donkey‐anti‐rabbit AlexaFluor 488 – ThermoFisherScientific A‐21206
Donkey‐anti‐mouse AlexaFluor 555 – ThermoFisherScientific A‐31570
DAPI – Sigma D9542 (10 µg mL^−1^)

### Confocal Microscopy and Analysis of Gap Junctional Puncta

Fixed and stained 3D gels (50–100 µL drops) were re‐positioned into 35 mm µ‐dishes (Ibidi) and imaged using Zeiss 7–10 confocal microscope. Approximately 50 µm stacks were obtained per image at system‐optimized inter‐slice intervals. Images were analyzed using ImageJ software, where gap junctional puncta were detected automatically using the “3D Object Counter” plugin. Size and intensity threshold of gap junctional puncta were determined using a 7 day‐matured picture as a guide and maintained throughout the experiment. Total fluorescence intensity was measured in the whole imaging field using the Integrated Density function following background subtraction. Nuclei per field were counted manually.

### Microinjection Dye Loading

Astrocyte monocultures were grown on 13 mm coverslips (VWR) for 5–7 days; on the day of injection, the cells were pre‐treated with 20 µM TAT‐GAP19 (Tocris) for 10 min prior to, and during microinjection to reduce dye uptake through Cx43‐containing hemichannels. A single cell was then microinjected with 0.5% lucifer yellow dye (Invitrogen) and 2% neurobiotin dye (Vector) in PBS using the FemtoJet microinjector system (Eppendorf) combined with FemtoTips II (Eppendorf). Injection pressure was set to 100 hPa for 0.1 s injection, constant pressure was kept at 10 hPa, and the needle was left in the injected cell for 5 min at room temperature. Cells on coverslip were then fixed in 4% PFA for 10 min and stained for further microscopic analysis. Images were analyzed in ImageJ software; injection areas were automatically thresholded and overlayed on the DAPI channel, after which the number of nuclei within the injection area were counted.

List of antibodies/dyes for microinjected area staining (all used at 1:500 final dilution):

Anti‐lucifer yellow rabbit – Invitrogen A‐5750
Cy3‐Streptavidin – Vector SA‐1300‐1

### Calcium Imaging

3D gels with astrocytes (50–100 µL at 8 × 10^6^ astrocytes mL^−1^) or 2D astrocytes (100 000 cells) cultured on MEAs were matured for 5 days prior to the recording, on the day of the recording—gently rinsed with PBS, and loaded with Fluo‐4 AM dye (5 µm, Invitrogen) and 0.04% pluronic F‐127 (Life Technologies) non‐ionic surfactant in Live Cell Imaging Solution (Invitrogen) for 1 h at 37 °C. The dye was then washed off, and cultures were allowed to acclimate for 1 h at 37 °C in the Neuronal Maintenance medium prior to re‐positioning into 35 mm µ‐dishes (re‐positioning step is applicable to 3D gels only) and recording with a spinning disk confocal microscope (Nikon) at 5 Hz sampling rate (0.2 s acquisition time per image) at 37 °C in a CO_2_‐controlled chamber. At least three independent cultures were sampled per condition. Δ*F*/*F*_0_ graphs were generated in Fiji software where cell bodies of interest were manually selected. Fluorescence intensity was measured at each cell body over time and normalized to its initial baseline fluorescence value.

### MEA Fabrication

For the AstroMEA device, six‐well MEAs comprised of PEDOT:PSS‐coated Au electrodes were fabricated according to a previously reported protocol.^[^ ^116^ ^]^ Briefly, the Au layer was patterned first on 5 cm × 5 cm glass substrates using lift‐off. PEDOT:PSS (containing 5% v/v ethylene glycol, 0.0015% v/v Dibenzyl sulfonic acid, and 1% v/v 3‐Glycidyloxypropyl‐trimethoxysilane) was spin‐coated on the substrates that were then baked at 120 °C for 1 h and subsequently soaked in de‐ionized water overnight. The PEDOT:PSS layer was patterned by photolithography and reactive ion etching. A chemical vapor‐deposited conformal Parylene C layer was used as the insulating layer and patterned by photolithography and reactive ion etching to expose the recording electrodes and contact pads. The resulting MEA had 60 electrodes (including ground electrodes) arranged in 6 groups of 9 recording/stimulating electrodes per group with 30 µm electrode diameter and electrode spacing of 200 µm.

For the calcium recording, 64 channel MEAs in a single well were used with electrode diameter of 100 µm and electrode spacing of 500 µm. The Au layer was patterned on 76 mm × 26 mm microscope slides using lift‐off. Parylene C layer was deposited as the insulating layer and patterned by photolithography and reactive ion etching to expose the recording electrodes and contact pads. The photoresist was left in place after etching and the process proceeded to the next PEDOT:PSS patterning step. PEDOT:PSS (containing 5% v/v ethylene glycol and 0.25% v/v 4‐dodecylbenzenesulfonic acid (DBSA), and 1% v/v of 3‐glycidyloxypropyl‐trimethoxysilane) was spin‐coated on the substrates that were then baked at 100 °C for 1 h. The PEDOT:PSS layer was patterned by photolithography and reactive ion etching, and after removing all the photoresist on the device, the device was soaked in de‐ionized water overnight.

Devices were sterilized with 70% ethanol and air‐dried prior to coating and cell seeding.

### AstroMEA Well‐Containing Overlay Fabrication

AstroMEA overlay was designed using Autodesk Fusion 360 (2.0) to fit the six‐well MEA dimensions. Starting from a solid round bar of polymethyl methacrylate (PMMA, acrylic), the cell culture wells were cut out by a milling machine (CNC).

### AstroMEA Recording and Stimulation Protocol

Recording and stimulation of the AstroMEA were performed using Intan RHS Stimulation/Recording system (Intan Technologies, USA) at 30 kHz sampling rate at 37 °C in a CO^2^‐controlled chamber. After 10 min of baseline recording, all electrodes in selected wells were stimulated with a theta‐burst^[^ ^54^ ^]^ charge‐balanced biphasic current stimulation which consisted of five pulses of 100 Hz stimulation repeated 10 times with 200 ms intervals, making a total of 50 pulses per 2 s‐long stimulation episode. Four independent devices containing biological replicates of astrocyte cultures with nine electrodes per well were included in the analysis, three to five stimulations spaced 10 min apart were delivered per device.

### Spike Detection and Raster Plot Generation

The raw recordings were band‐pass filtered (300 – 3000 Hz) first and spikes were detected from the filtered signal based on an amplitude threshold. A threshold of –5 times the standard deviation (calculated for each electrode based on the level of noise) was chosen. The time instances of the threshold crossing were considered for creating spike‐trains from each electrode which were used to compute the inter‐spike interval histogram (with 2 ms bins) and raster plots. 2 ms‐wide signals around the spike minima were extracted as spike waveforms from individual electrodes. The analyses were carried out by custom‐written python scripts.

### Statistical Analysis

For evaluation of Cx43 puncta and total fluorescence in astrocytic monocultures at several timepoints, differences were analyzed using a one‐way analysis of variance (ANOVA) with a Tukey post hoc test (α = 0.05). A paired t‐test (α = 0.05) was used to detect changes between the baseline signaling and signaling at the 0–30 s interval post‐theta burst stimulus (after the exclusion of the stimulus period of 2 s plus 2 ms directly following the stimulus to avoid artifacts). Normality of the data distribution was typically measured using Shapiro–Wilk test; but normality of the data distribution was not taken into consideration following the previously published argument since all datasets contained *n* > 50 measurements.^[^ ^117^ ^]^ No pre‐processing of data was employed; analysis was performed in R; sample sizes are indicated in relevant figures and text. Data presented graphically as mean ± SEM. In all cases, significance was defined as *p* ≤ 0.05.

### Ethics Approval Statement

All studies involving rat brain cell culture derived from newborn rat pups were approved by the Animal Welfare Ethical Review Body at the University of Cambridge as a schedule 1 non‐regulated (not requiring a personal or project license) procedure.

## Conflict of Interest

The authors declare no conflict of interest.

## Author Contributions

N.H. formulated the hypothesis, designed the AstroMEA device, cultured the cells, conducted the staining and live calcium recordings, wrote the paper, and generated the figures. B.H. and M.I. manufactured the MEAs; S.M. and R.J.M. performed the electrophysiological recordings and conducted the electrophysiological analysis; Y.L.Y. contributed to the cell culture and device design; A.C.‐L. assisted with the device design; N.A.E. manufactured the AstroMEA wells; S.R. conducted the astrocyte culture purity analysis; T.P.J.K., M.B., G.G.M., and M.R.N.K. oversaw the project and provided substantial additions and edits. All authors contributed to multiple parts of the paper as well as the overall content.

## Supporting information

Supporting Information

Supporting Information

Supporting Information

## Data Availability

The data that support the findings of this study are available from the corresponding author upon reasonable request.
