# Supplementary material for: Electrophysiological In Vitro Study of Long‐Range Signal Transmission by Astrocytic Networks
Source: Adv Sci (Weinh). 2023 Jul 23;10(29):2301756. doi: 10.1002/advs.202301756 (PMC10582426; doi:10.1002/advs.202301756)
Supplement: Supplementary file 1 — Supporting Information [file ADVS-10-2301756-s002.pdf]

ADVANCED SCIENCE
Open Access

Supporting Information

for *Adv. Sci.*, DOI 10.1002/advs.202301756

Electrophysiological In Vitro Study of Long-Range Signal Transmission by Astrocytic Networks

Nataly Hastings, Yi-Lin Yu, Botian Huang, Sagnik Middya, Misaki Inaoka, Nadia A. Erkamp, Roger J. Mason, Alejandro Carnicer-Lombarte, Saifur Rahman, Tuomas P. J. Knowles, Manohar Bance, George G. Malliaras and Mark R. N. Kotter*

Electrophysiological *In Vitro* Study of Long-Range Signal Transmission by Astrocytic Networks

Nataly Hastings*, Yi-Lin Yu, Botian Huang, Sagnik Middya, Misaki Inaoka, Nadia A. Erkamp, Roger J. Mason, Alejandro Carnicer-Lombarte, Saifur Rahman, Tuomas P.J. Knowles, Manohar Bance, George G. Malliaras, and Mark R.N. Kotter

Supplementary Information

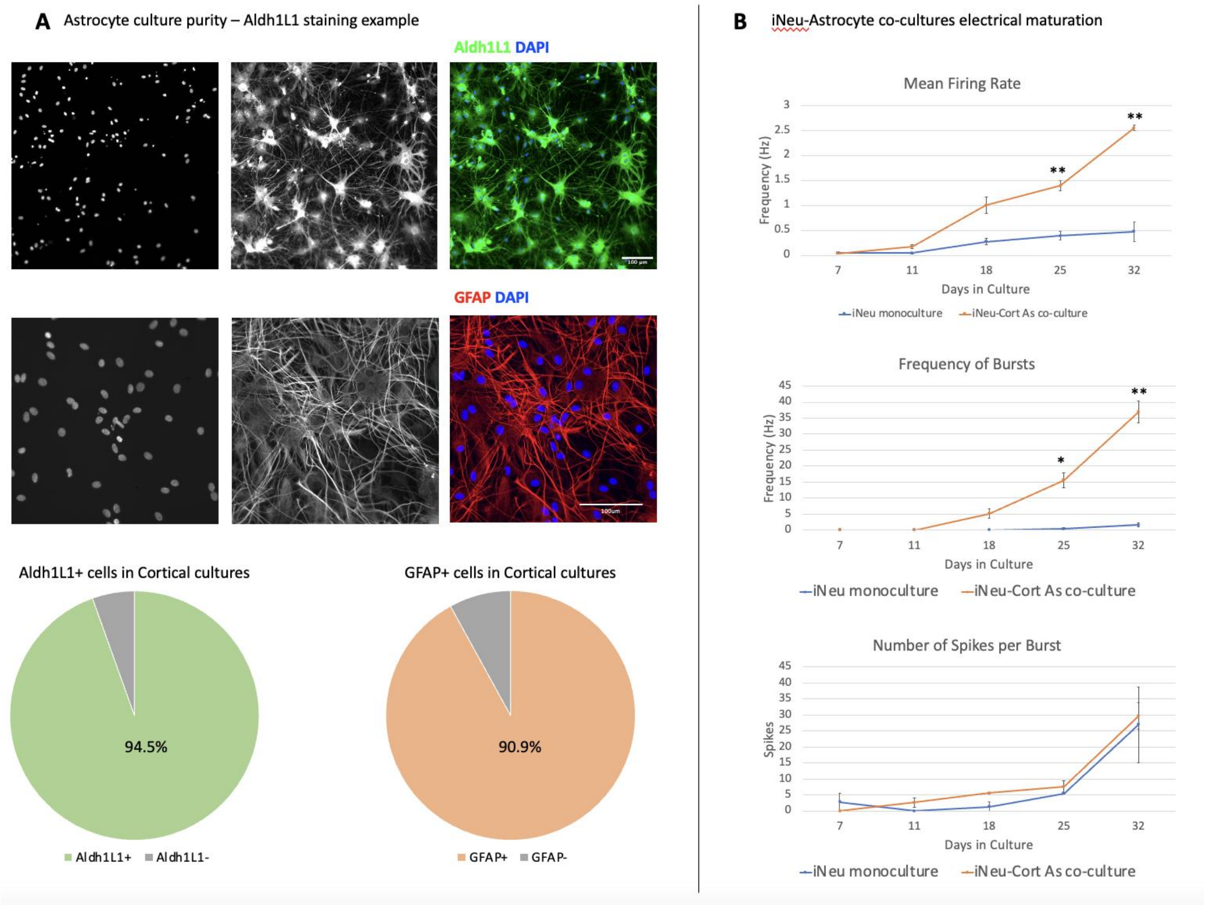

Supplementary Figure 1. **Characterization of astrocyte monocultures and iNeuron (iNeu)-astrocyte co-cultures.** A: Cortical rat brain (cortical) cell cultures enriched for astrocytes exhibit detectable astrocyte-lineage markers Aldh1L1 and GFAP in over 90% of cells. Scale bar: 100 μm, *n*=3 cultures per analysis. B: Time course of electrical maturation of iNeu-astrocyte co-cultures (1:1 ratio) or iNeu monocultures on 48 plate MEAs with 16 electrodes per well (Axion Biosystem, USA). Spike threshold set to 5 times the standard deviation; a burst event was defined as a train of at least 5 spikes occurring with no more than 100 ms inter-spike intervals. *n*=4. Error bars: SEM. P-values were calculated using a using a repeated measures analysis of variance (ANOVA) with a Šídák's multiple comparisons test. *p<0.05, **p<0.005.

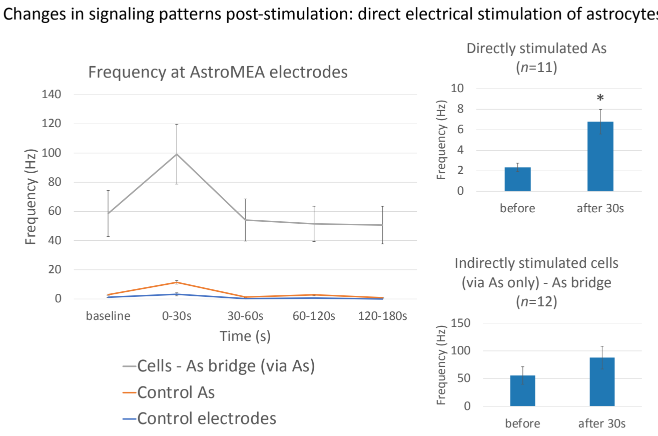

Supplementary Figure 2. **Signal transmission via an astrocytic network after stimulation of an astrocyte monoculture.** Changes in signaling patters after a theta-burst stimulation. iNeuron (iNeu)-astrocyte (As) co-cultures which were stimulated indirectly via an astrocytic bridge following a direct theta-burst stimulation of astrocyte monocultures, showed a slightly increased action potential firing patterns within 30 s directly following the stimulus compared to the baseline firing rate. Error bars: SEM. P-values were measured using a Wilcoxon Signed Rank test. *p<0.05.

Supplementary Video 1: **Live calcium wave in a 3D astrocytic network induced by ATP.** Calcium wave spread in a 3D astrocytic network in real time after 100 μM ATP application (source of ATP was originally in the top right corner of the imaging field).

Supplementary Video 2: **Live calcium wave in a 2D astrocytic network induced by an electrical theta-burst.** Calcium waves are initiated in cultured astrocytes in real time following a charge-balanced biphasic current stimulation which consisted of 5 pulses of 100 Hz stimulation repeated 10 times with 200 ms intervals, making a total of 50 pulses per 2 s-long stimulation episode.

Supplementary Experimental Section / Methods

1. 2D culture immunostaining

Astrocytic culture purity was established via immunostaining against astrocytic markers. 2D cultures of astrocytes on coverslips were fixed with 4% PFA for 5 min at room temperature, after which coverslips were rinsed in PBS three times and incubated in blocking and permeabilization buffer containing 5% donkey serum (Abcam) and 0.5% tritonX-100 (Sigma) in PBS for 1 h at room temperature. Primary and secondary antibody incubations were conducted in a staining buffer containing 1% donkey serum and 0.1% tritonX-100 for 1 h at room temperature on a rocking shaker each with 3 x 10 min PBS washes in between. Nuclei were visualized with DAPI (ThermoFisherScientific) added to the secondary antibody solution. At least 3 independent cultures were assessed.

List of antibodies (all used at 1:500 final dilution):
Anti-Aldh1L1 rabbit – Abcam ab190298
Anti-GFAP chicken – GeneTex GTX85454

2. Neuronal monoculture vs co-culture electrical maturation comparison

Neuronal monocultures or iNeu co-cultured with cortical astrocytes were plated on CytoView 48 plate MEAs (Axion Biosystem, USA) at a 1:1 ratio with a total number of

100,000 cells per well (50,000 astrocytes + 50,000 iNeu in co-cultures, or 100,000 iNeu in monocultures). Each MEA plate well contained 16 electrodes. Recordings were taken on day 7 post-plating, followed by day 11, and every 7 days thereafter until day 32 post-plating using a Maestro Pro (Axion Biosystem), sampled at a frequency of 12.5 kHz. Each recording event lasted 20 min. Spike detection was performed using the peak detection adaptive threshold method with a variable threshold set to 5 times the standard deviation to detect the maximum amplitude. A burst event was defined as a train of at least 5 spikes occurring with no more than 100 ms inter-spike intervals. 4 independent co-cultures were included in the analysis.

3. Astrocytic monocultures in AstroMEA wells

Astrocyte-iNeurone co-cultures and 3D gels with astrocytes were plated as described in the main Methods / Experimental Section. For the cortical astrocyte monocultures, 50,000 cells were plated on top of electrodes in 2D at the same time as co-cultures, after which the processing of the AstroMEA was identical to the timeline shown in Figure 5B.

4. Statistical analysis

For electrical maturation of mono- vs co-cultures, differences between cell culture conditions at various timepoints were analyzed using a repeated measures analysis of variance (ANOVA) with a Šídák's multiple comparisons test (α=0.05). A Wilcoxon Signed rank test (α=0.05) was used to detect changes between the baseline signaling and signaling at the 0-30 s interval post-theta burst stimulus (after the exclusion of the stimulus period of 2 s plus 2 ms directly following the stimulus to avoid artifacts) in the iNeu-astrocyte co-cultures following the direct electrical stimulation of the astrocyte monocultures. Normality of the data distribution was measured using Shapiro-Wilk test, and since the data were not normally distributed in case of the signal transmission via an astrocytic network after stimulation of an astrocyte monoculture analysis with the n<50 measurements,[113] a non-parametric alternative to a paired t-test was utilized. No pre-processing of data was employed; analysis was performed in R; sample sizes are indicated in relevant figures and text. Data presented graphically as mean $\pm$ SEM. In all cases, significance was defined as $p \leq 0.05$.
